# Incomplete Family History and Meeting Algorithmic Criteria for Genetic Evaluation of Hereditary Cancer

**DOI:** 10.1001/jamanetworkopen.2025.39870

**Published:** 2025-10-28

**Authors:** Adrian Harris, Jemar R. Bather, Richard L. Bradshaw, Kensaku Kawamoto, Guilherme Del Fiol, Wendy K. Kohlmann, Daniel Chavez-Yenter, Rachel Monahan, Rachelle L. Chambers, Meenakshi Sigireddi, Melody S. Goodman, Kimberly A. Kaphingst

**Affiliations:** 1 Center for Anti-racism, Social Justice & Public Health, New York University School of Global Public Health, New York, New York; 2 Department of Biostatistics, New York University School of Global Public Health, New York, New York; 3 Department of Biomedical Informatics, Spencer Fox Eccles School of Medicine, University of Utah, Salt Lake City; 4 Huntsman Cancer Institute, University of Utah, Salt Lake City; 5 Clinical Cancer Genetics Service, Veterans Affairs Medical Center National TeleOncology, Durham, North Carolina; 6 Division of Hematology-Oncology, Perelman School of Medicine, University of Pennsylvania, Philadelphia; 7 Department of Medical Ethics and Health Policy, Perelman School of Medicine, University of Pennsylvania, Philadelphia; 8 Perlmutter Cancer Center, NYU Langone Health, New York, New York; 9 Department of Communication, University of Utah, Salt Lake City

## Abstract

**Importance:**

Incomplete electronic health record (EHR) documentation may limit the effectiveness of clinical decision support (CDS) algorithms designed to identify patients eligible for hereditary cancer genetic evaluation.

**Objectives:**

To determine whether a CDS algorithm can identify patients who meet criteria for hereditary cancer genetic evaluation when family history data are incompletely documented in the EHR, and to examine whether data missingness is associated with identification patterns across patient subgroups.

**Design, Setting, and Participants:**

This cross-sectional study analyzed EHR data extracted in December 2020 from 2 large US health care systems: University of Utah Health (UHealth) and NYU Langone Health (NYULH). Eligible patients were adults aged 25 to 60 years who visited a primary care clinic within the previous 3 years and had some EHR documentation of cancer family history. Data analysis was conducted in August 2024.

**Exposures:**

Patient demographic factors (age, sex, race and ethnicity, and language preference) and cancer family history characteristics (number of cancer history records, number of affected first- and second-degree relatives, relatives with rising mortality cancers, presence of hereditary cancer-related terms in comments, and completeness of documentation).

**Main Outcomes and Measures:**

The primary outcome was meeting at least 1 CDS algorithm criterion for genetic evaluation of hereditary cancer risk based on National Comprehensive Cancer Network guidelines. Missing data patterns were assessed using the Little missing completely at random test, with analyses conducted using complete case analysis and multiple imputation.

**Results:**

This study included 157 207 patients: 55 918 from UHealth and 101 289 from NYULH. Their mean (SD) age was 43.5 (9.8) years, and most (65.7%) were female. A total of 5607 UHealth patients (10.0%) and 10 375 NYULH patients (10.2%) met CDS criteria for genetic evaluation. At UHealth, data appeared to be missing completely at random (χ^2^_39_ = 39.09; *P* = .47), and complete case compared with multiple imputation analyses yielded similar results. At NYULH, data were not missing completely at random (χ^2^_55_ = 914.89; *P* < .001). Compared with multiple imputation, complete case analysis produced different association magnitudes for older age and having relatives with rising mortality cancers, suggesting bias when excluding incomplete records.

**Conclusions and Relevance:**

In this cross-sectional study, the magnitude of the association between incomplete family history documentation and identification of patients eligible for hereditary cancer genetic evaluation depended on whether data were missing randomly or systematically. These findings suggest that health care organizations implementing CDS algorithms should assess their specific missing data patterns and consider tailored approaches to handling incomplete family history information to ensure equitable identification of all patients who could benefit from genetic evaluation services.

## Key Points

**Question:**

Can a clinical decision support algorithm identify patients who meet criteria for hereditary cancer genetic evaluation when family history data are incompletely documented in the electronic health record, and is data missingness associated with identification patterns in patient subgroups?

**Findings:**

In this cross-sectional study of 157 207 patients from 2 US health care systems who had documentation of cancer family history, 10% met genetic evaluation criteria. Where data were missing randomly, incomplete documentation was not associated with patient identification; however, when data were missing systematically, older patients and those with relatives with rising mortality cancers were significantly more likely to be identified when incomplete records were excluded.

**Meaning:**

These findings suggest that health care systems should assess their specific missing data patterns when implementing clinical decision support algorithms.

## Teaser

This cross-sectional study evaluates whether a clinical decision support algorithm can identify patients with incomplete electronic health record documentation of family history who are eligible for hereditary cancer genetic evaluation.

## Introduction

Advances in technology may affect individuals at many stages along the cancer continuum.^1^ For example, advances in machine learning and algorithmic approaches have improved cancer detection through diagnostic imaging and treatment selection.^2^ However, there can be access barriers to these technologies at multiple stages. Cancer prevention and screening strategies are often limited by technological barriers as well as by socioeconomic barriers and access to health care.^3,4^ Another important barrier is the limited availability of comprehensive data from multiple sources, including health care practitioners, family members, and patients themselves. Incomplete electronic health records (EHRs) can limit the clinical utility and reach of technological advances and research that require specific data inputs.

Data completeness is affected by several critical factors. First, the US lacks standardized data collection processes across health care systems.^5^ Second, underrepresented groups, including Black and Hispanic individuals and older adults, may be less willing to share sensitive medical information due to previous negative health care experiences.^5,6^ Patient mistrust of the health care system,^7^ stemming from prior interactions with medical professionals, can substantially affect data quality and completeness. Third, cultural differences in sharing private health information may hinder communication of cancer family history among relatives.^5,6^ These factors collectively influence the patient data available for analysis, which affects the ability of EHR-based technologies, such as clinical decision support (CDS) algorithms that can identify appropriate candidates for interventions.

Differences in data completeness across groups can create systematic gaps in datasets.^8^ If unaddressed, these differences may cause specific groups to receive disproportionately less benefit from new technologies that rely on the data^8^; for example, individuals not represented in samples may miss opportunities for preventative care.^9,10^ These data completeness challenges are particularly significant in the context of hereditary cancer syndromes, where evidence-based clinical criteria exist to identify patients without a personal history of cancer who should receive genetic evaluation based on specific constellations of cancer family histories.^11,12,13^ EHR-based technologies, such as CDS algorithms, are important for identifying patients who meet these criteria, as studies show physicians may lack awareness of genetic foundations for familial disorders, resulting in missed referrals for genetic testing.^14,15^ However, such algorithms may be substantially affected by missing data in the EHR. Currently, there is no clear framework for handling missing data necessary to identify patients eligible for genetic evaluation based on family history, resulting in a critical knowledge gap. Therefore, in this study, we assessed whether a CDS algorithm can identify patients who meet criteria for hereditary cancer genetic evaluation when family history data are incompletely documented in the EHR, and we examined whether data missingness is associated with identification patterns across patient subgroups.

## Methods

### Study Design and Setting

This cross-sectional study was conducted within the Broadening the Reach, Impact, and Delivery of Genetic Services (BRIDGE) trial.^16,17^ To address the need for improved identification of patients eligible for genetic evaluation, the team developed GARDE (Genetic Cancer Risk Detector), a standards-based population health platform.^18,19^ GARDE uses rule-based algorithms to identify individuals who meet guideline-based criteria for genetic evaluation for hereditary breast and colorectal syndromes based on cancer family history information in the EHR.^19^ The algorithms were adapted from the National Comprehensive Cancer Network (NCCN) guidelines for genetic evaluation of familial cancers.^18^ The GARDE algorithms required complete information on specific data elements (eg, cancer type, relative type, and age of cancer onset) to identify eligible individuals based on these criteria (eg, first- or second-degree relative with breast cancer who had an age of onset <45 years). GARDE evaluated adult patients aged 25 to 60 years who had visited a primary care clinic within the previous 3 years. This age range was selected to enable unaffected patients to benefit from recommended cancer prevention and screening steps following a cancer genetic evaluation.^16^ We analyzed data from 2 large health care systems: University of Utah Health (UHealth) and NYU Langone Health (NYULH). UHealth provides care to more than 1 million patients spanning 6 states, while NYULH serves more than 8 million patients across 300 ambulatory sites and affiliate hospitals. These health care systems present valuable comparison opportunities due to their differences in patient demographics, organizational structures, and geographic service areas.

The University of Utah Institutional Review Board approved this study on behalf of UHealth and NYULH; the procedures described herein were approved with a waiver of informed consent due to the use of deidentified data. We adhered to the Strengthening the Reporting of Observational Studies in Epidemiology (STROBE) reporting guideline.

### Analytic Sample

In December 2020, the BRIDGE study team extracted EHR data for 522 105 patients: 144 484 from UHealth and 377 621 from NYULH (Figure). For this analysis, we applied 2 exclusion criteria: (1) age younger than 25 or older than 60 years and (2) absence of cancer family history information. For UHealth, we excluded 958 patients due to missing age and 87 608 patients (61.3%) due to missing cancer family history, resulting in 55 918 eligible patients. Of these UHealth patients, 54 982 had complete data for the complete case analysis, whereas 936 had partial missing data and were retained only for multiple imputation analysis. For NYULH, we excluded 36 074 patients due to missing age and 240 258 patients (73.2%) due to missing cancer family history, resulting in 101 289 eligible patients. Of these NYULH patients, 82 093 had complete data for the complete case analysis, whereas 19 196 had partial missing data and were retained only for multiple imputation analysis.

**Figure.**
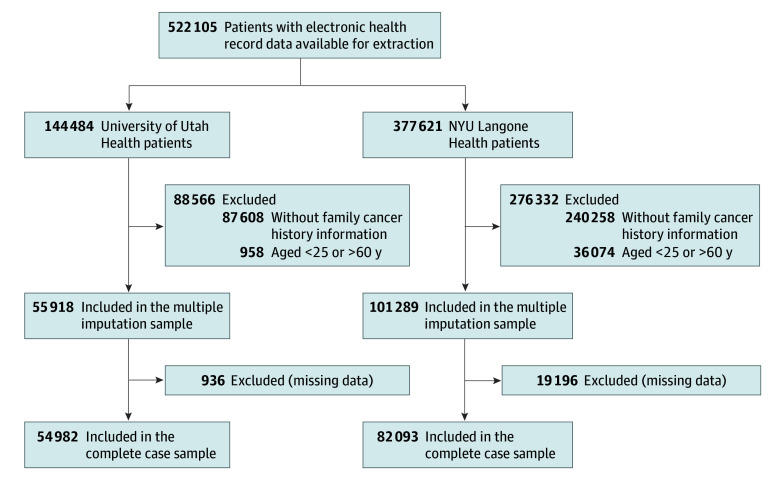
Selection of Patients From University of Utah Health and NYU Langone Health

### Dependent Variable

The outcome of interest was whether the patient met at least 1 of the GARDE algorithm criteria for genetic evaluation for hereditary cancer. The GARDE algorithms, adapted from NCCN guidelines, are presented in Table 1. Examples of these criteria included having a first-degree or second-degree relative with ovarian or pancreatic cancer, or being of Ashkenazi Jewish heritage with a family history of breast, ovarian, prostate, or pancreatic cancer.^18,19^

**Table 1.** GARDE Algorithms Adapted From National Comprehensive Cancer Network Guidelines for Genetic Evaluation of Hereditary Risk for Breast and Colorectal Cancer

Indication	Algorithm
Breast cancer genetic evaluation	Problem list or family history documentation of *BRCA1/2*, *CHEK2*, *ATM*, *PALB2*, *TP53*, *PTEN*, *CDH1*, Cowden syndrome, or Li-Fraumeni syndrome
	Relative (first or second degree) diagnosed with breast cancer before age 45 y
	Relative (first or second degree) with ovarian or pancreatic cancer
	Male relative (first or second degree) with breast cancer
	≥3 Relatives (first or second degree) on same lineage with breast or prostate cancer
	Ashkenazi Jewish heritage plus family history of breast, ovarian, prostate, or pancreatic cancer
Colorectal cancer genetic evaluation	Problem list or family history documentation of *MLH1*, *MSH2*, *MSH6*, *PMS2*, *EPCAM*, Lynch syndrome, hereditary nonpolyposis colorectal cancer, familial adenomatous polyposis, *APC*, *MYH*, *MUTYH*, or serrated polyposis
	Relative (first or second degree) with colorectal cancer diagnosed before age 50 y
	Relative (first or second degree) with endometrial cancer diagnosed before age 50 y
	≥3 Relatives (first or second degree) on same lineage (regardless of age of onset) with bladder, brain, colorectal, endometrial, gastric, kidney, ovarian, pancreatic, small bowel, ureteral, or urethra cancer

Abbreviation: GARDE, Genetic Cancer Risk Detector.

### Independent Variables

#### Demographic Factors

We sourced the following demographic variables from each patient’s EHR: age (categorized for analysis: 25-29, 30-44, or 45-60 years), sex (female or male), language preference (English or Spanish), and race and ethnicity (Hispanic, non-Hispanic Black, non-Hispanic White, and non-Hispanic other race or ethnicity [including American Indian or Alaska Native, Asian, Middle Eastern or North African, and Native Hawaiian or Other Pacific Islander]). Data on race and ethnicity were collected because patients from underrepresented groups are more likely to withhold sensitive medical information owing to previous negative experiences with health care.

#### Family History of Cancer

Information on cancer family history included the number of cancer family history records (ie, the number of instances of family history of cancer recorded as structured data in a patient’s EHR), the number of first- and second-degree relatives with cancer history records (ie, the number of instances of family history of cancer for a first- or second-degree relative recorded in a patient’s EHR), and the number of relatives with cancers with rising mortality (ie, defined instances of family history of cancers showing increasing mortality rates from 2016 to 2020): specifically brain cancer, pancreatic cancer, uterine cancer, liver cancer, and throat cancer.^20^ We included the latter measure because the rising mortality trends of those cancers may have increased clinical attention and documentation.

The EHR’s family history section included free-text comment fields that potentially contained information absent from structured fields. Using a string-matching algorithm,^21^ we searched these comments for hereditary cancer-related terms spanning 3 categories: (1) specific gene variants (eg, *BRCA1*, *BRCA2*, *MLH1*, or *MSH2*), (2) hereditary cancer syndromes (eg, Lynch syndrome, Li-Fraumeni syndrome, or Cowden syndrome), and (3) relevant cancer characteristics (eg, triple negative, hormone negative, or metastatic). Patients were assigned “yes” if any of these data elements were found in their comments and “no” otherwise.

We constructed a comprehensiveness score to measure the completeness of cancer family history documentation in structured EHR fields. This score was based on 3 data elements from family history records: relative relationship, cancer type, and age of cancer onset.^22^ Patients received a score of “2” if 2 elements were documented (relative relationship and cancer type) or “3” if all 3 elements were documented (relative relationship, cancer type, and age of onset).

### Statistical Analysis

We generated descriptive statistics for all variables, including counts and proportions for categorical measures and means (SDs) for continuous variables. We assessed bivariate associations between each independent variable and the outcome using standardized mean differences (SMDs) with 95% CIs.

Prior to multivariable analysis,^23^ we evaluated missing data patterns at each health care system (UHealth and NYULH) using the Little missing completely at random (MCAR) test.^24^ We estimated multivariable associations using modified Poisson regression,^25,26^ fitting separate models for each health care system. We conducted analyses under both complete case analysis and multiple imputation by chained equations (mice package in R),^27^ with imputation methods tailored to variable type: logistic regression for sex and language preference, multinomial logistic regression for race and ethnicity, and ordinal logistic regression for age. Multiple imputation results were pooled across 20 iterations using Rubin rules.^28^ All models yielded adjusted prevalence ratios (APRs) with 95% CIs.

We conducted all data analyses using R, version 4.5.1 (R Project for Statistical Computing), with statistical significance defined as 2-tailed *P* < .05. Data analysis was conducted in August 2024.

## Results

### Patient Characteristics

This study included 157 207 patients (mean [SD] age, 43.5 [9.8] years); 65.7% were female and 33.9% were male (sex was missing for 0.3%). Table 2 and Table 3 present patient characteristics for UHealth (n = 55 918) and NYULH (n = 101 289), respectively, stratified by whether patients met GARDE algorithm criteria for cancer genetic evaluation. A total of 5607 UHealth patients (10.0%) and 10 375 NYULH patients (10.2%) met these criteria. The overall patient populations differed by age (≥30 years: 84.9% for UHealth compared with 95.1% for NYULH) and by some racial and ethnic categories (UHealth and NYULH patients identified as Hispanic [10.0% and 2.0%, respectively], non-Hispanic Black [1.2% and 10.9%, respectively], non-Hispanic White [80.4% and 61.6%, respectively], or non-Hispanic other race or ethnicity [6.7% and 6.7%, respectively]; these data were missing for 1.7% and 18.8% of patients, respectively). When comparing patients who met criteria with those who did not, both sites showed meaningful differences (SMD >0.10) in sex distribution, cancer family history metrics (number of records, affected first- and second-degree relatives, and relatives with rising mortality cancers), presence of a hereditary cancer-related term in family history comments, and comprehensiveness scores (Tables 2 and 3). NYULH showed 2 additional areas of meaningful difference: age and language preference (Table 3).

**Table 2.** University of Utah Health Patient Characteristics, Overall and Stratified by Meeting at Least 1 GARDE Algorithm Criterion for Genetic Evaluation of Hereditary Cancer Risk^a^

Characteristic	Overall population (n = 55 918)	Patients meeting ≥1 GARDE algorithm criterion		Standardized mean difference (95% CI)
		No (n = 50 311)	Yes (n = 5607)	
Age, y				
25-29	8458 (15.1)	7577 (15.1)	881 (15.7)	0.08 (0.05-0.11)
30-44	27 680 (49.5)	24 748 (49.2)	2932 (52.3)	
45-60	19 780 (35.4)	17 986 (35.7)	1794 (32.0)	
Sex				
Female	37 415 (66.9)	32 904 (65.4)	4511 (80.5)	0.34 (0.32-0.37)
Male	18 500 (33.1)	17 404 (34.6)	1096 (19.5)	
Missing	3 (<0.1)	3 (<0.1)	0	
Race and ethnicity				
Hispanic				0.03 (0.00-0.06)
Non-Hispanic Black	658 (1.2)	588 (1.2)	70 (1.2)	
Non-Hispanic White	44 979 (80.4)	40 496 (80.5)	4483 (80.0)	
Non-Hispanic other^b^	3766 (6.7)	3390 (6.7)	376 (6.7)	
Missing	932 (1.7)	849 (1.7)	83 (1.5)	
Language preference				
English	54 166 (96.9)	48 702 (96.8)	5464 (97.4)	0.04 (0.01-0.07)
Spanish	1750 (3.1)	1607 (3.2)	143 (2.6)	
Missing	2 (<0.1)	2 (<0.1)	0	
No. of family cancer history records				
1	26 794 (47.9)	25 350 (50.4)	1444 (25.8)	0.61 (0.58-0.63)
2	15 034 (26.9)	13 539 (26.9)	1495 (26.7)	
≥3	14 090 (25.2)	11 422 (22.7)	2668 (47.6)	
No. of first-degree relatives with cancer history				
0	24 941 (44.6)	22 836 (45.4)	2105 (37.5)	0.30 (0.27-0.33)
1	20 545 (36.7)	18 728 (37.2)	1817 (32.4)	
≥2	10 432 (18.7)	8747 (17.4)	1685 (30.1)	
No. of second-degree relatives with cancer history				
0	23 685 (42.4)	22 123 (44.0)	1562 (27.9)	0.48 (0.45-0.51)
1	20 575 (36.8)	18 725 (37.2)	1850 (33.0)	
≥2	11 658 (20.8)	9463 (18.8)	2195 (39.1)	
No. of first- or second-degree relatives with a rising mortality cancer				
0	52 953 (94.7)	48 619 (96.6)	4334 (77.3)	0.60 (0.57-0.63)
1	2706 (4.8)	1576 (3.1)	1130 (20.2)	
≥2	259 (0.5)	116 (0.2)	143 (2.6)	
Hereditary cancer-related term found in family history section comment				
No	55 554 (99.3)	50 074 (99.5)	5480 (97.7)	0.15 (0.13-0.18)
Yes	364 (0.7)	237 (0.5)	127 (2.3)	
Comprehensiveness score				
2	53 188 (95.1)	48 394 (96.2)	4794 (85.5)	0.38 (0.35-0.40)
3	2730 (4.9)	1917 (3.8)	813 (14.5)	

Abbreviation: GARDE, Genetic Cancer Risk Detector.

^a^ Unless otherwise indicated, values are presented as No. (%) of patients.

^b^ Includes American Indian or Alaska Native, Asian, Middle Eastern or North African, and Native Hawaiian or Other Pacific Islander.

**Table 3.** NYU Langone Health Patient Characteristics, Overall and Stratified by Meeting at Least 1 GARDE Algorithm Criterion for Genetic Evaluation of Hereditary Cancer Risk^a^

Characteristic	Overall population (n = 101 289)	Patients meeting ≥1 GARDE algorithm criterion		Standardized mean difference (95% CI)
		No (n = 90 914)	Yes (n = 10 375)	
Age, y				
25-29	4565 (4.5)	4240 (4.7)	325 (3.1)	0.15 (0.13-0.17)
30-44	43 116 (42.6)	38 266 (42.1)	4850 (46.7)	
45-60	53 142 (52.5)	47 942 (52.7)	5200 (50.1)	
Missing	466 (0.5)	466 (0.5)	0	
Sex				
Female	65 938 (65.1)	58 054 (63.9)	7884 (76.0)	0.28 (0.26-0.30)
Male	34 848 (34.4)	32 361 (35.6)	2487 (24.0)	
Missing	503 (0.5)	499 (0.5)	4 (<0.1)	
Race and ethnicity				
Hispanic	2028 (2.0)	1881 (2.1)	147 (1.4)	0.06 (0.04-0.08)
Non-Hispanic Black	11 071 (10.9)	9962 (11.0)	1109 (10.7)	
Non-Hispanic White	62 359 (61.6)	55 936 (61.5)	6423 (61.9)	
Non-Hispanic other^b^	6824 (6.7)	6160 (6.8)	664 (6.4)	
Missing	19 007 (18.8)	16 975 (18.7)	2032 (19.6)	
Language preference				
English	95 833 (94.6)	85 693 (94.3)	10 140 (97.7)	0.18 (0.16-0.20)
Spanish	4652 (4.6)	4433 (4.9)	219 (2.1)	
Missing	804 (0.8)	788 (0.9)	16 (0.2)	
No. of cancer family history records				
1	64 753 (63.9)	60 771 (66.8)	3982 (38.4)	0.66 (0.64-0.68)
2	23 566 (23.3)	20 552 (22.6)	3014 (29.1)	
≥3	12 970 (12.8)	9591 (10.5)	3379 (32.6)	
No. of first-degree relatives with cancer history				
0	43 125 (42.6)	38 583 (42.4)	4542 (43.8)	0.24 (0.22-0.26)
1	46 880 (46.3)	42 891 (47.2)	3989 (38.4)	
≥2	11 284 (11.1)	9440 (10.4)	1844 (17.8)	
No. of second-degree relatives with cancer history				
0	45 159 (44.6)	42 485 (46.7)	2674 (25.8)	0.58 (0.55-0.60)
1	38 708 (38.2)	34 825 (38.3)	3883 (37.4)	
≥2	17 422 (17.2)	13 604 (15.0)	3818 (36.8)	
No. of first- or second-degree relatives with a rising mortality cancer				
0	88 256 (87.1)	81 457 (89.6)	6799 (65.5)	0.61 (0.59-0.63)
1	12 052 (11.9)	8873 (9.8)	3179 (30.6)	
≥2	981 (1.0)	584 (0.6)	397 (3.8)	
Hereditary cancer-related term found in family history section comment				
No	100 278 (99.0)	90 303 (99.3)	9975 (96.1)	0.22 (0.19-0.24)
Yes	1011 (1.0)	611 (0.7)	400 (3.9)	
Comprehensiveness score				
2	90 235 (89.1)	82 354 (90.6)	7881 (76.0)	0.40 (0.38-0.42)
3	11 054 (10.9)	8560 (9.4)	2494 (24.0)	

Abbreviation: GARDE, Genetic Cancer Risk Detector.

^a^ Unless otherwise indicated, values are presented as No. (%) of patients.

^b^ Includes American Indian or Alaska Native, Asian, Middle Eastern or North African, and Native Hawaiian or Other Pacific Islander.

### Missingness and Associations With Meeting GARDE Algorithm Criteria

The MCAR test results differed between sites. At UHealth, the MCAR test showed no evidence that missing data patterns were systematically related to observed measures (χ^2^_39_ = 39.09; *P* = .47); at NYULH, the test indicated that missingness depended on observed values (χ^2^_55_ = 914.89; *P* < .001). These findings informed the interpretation of the multivariable results in Table 4. For UHealth, where data were likely to be MCAR, complete case and multiple imputation analyses yielded similar APRs and 95% CIs across all independent variables. Meeting criteria at UHealth was associated with female sex (compared with male sex: APR, 1.61 [95% CI, 1.51-1.71]), Hispanic ethnicity (compared with non-Hispanic White race: APR, 1.17 [95% CI, 1.07-1.27]), higher cancer family history metrics (eg, ≥2 compared with 0 first-degree relatives with cancer history: APR, 1.64 [95% CI, 1.52-1.78]), presence of a hereditary cancer-related term in family history comments (compared with no presence: APR, 2.00 [95% CI, 1.71-2.33]), and higher comprehensiveness score (3 compared with 2: APR, 3.52 [95% CI, 3.29-3.77]). Conversely, not meeting criteria at UHealth was associated with older age (45-60 compared with 25-29 years: APR, 0.82 [95% CI, 0.76-0.88]) and Spanish language preference (compared with English: APR, 0.83 [95% CI, 0.70-0.98]).

**Table 4.** Comparison of Complete Case and Multiple Imputation Analyses of Factors Associated With Meeting Genetic Evaluation Criteria for Hereditary Cancer Risk, Stratified by Health Care System

Characteristic	Patients meeting ≥1 GARDE algorithm criterion for genetic evaluation of hereditary cancer risk, APR (95% CI)			
	University of Utah Health		NYU Langone Health	
	Complete case	Multiple imputation^a^	Complete case	Multiple imputation
Age, y				
25-29	1 [Reference]	1 [Reference]	1 [Reference]	1 [Reference]
30-44	0.97 (0.90-1.04)	0.96 (0.90-1.03)	1.58 (1.40-1.78)	1.46 (1.31-1.62)
45-60	0.81 (0.75-0.87)	0.82 (0.76-0.88)	1.46 (1.29-1.65)	1.36 (1.22-1.51)
Sex				
Female	1.60 (1.51-1.71)	1.61 (1.51-1.71)	1.23 (1.18-1.29)	1.27 (1.22-1.32)
Male	1 [Reference]	1 [Reference]	1 [Reference]	1 [Reference]
Race and ethnicity				
Hispanic	1.16 (1.07-1.27)	1.17 (1.07-1.27)	0.81 (0.69-0.95)	0.81 (0.69-0.94)
Non-Hispanic Black	1.22 (0.98-1.52)	1.22 (0.98-1.52)	1.02 (0.96-1.08)	1.01 (0.96-1.08)
Non-Hispanic White	1 [Reference]	1 [Reference]	1 [Reference]	1 [Reference]
Non-Hispanic other^b^	1.09 (0.99-1.20)	1.09 (0.99-1.20)	0.96 (0.89-1.03)	0.96 (0.90-1.03)
Language preference				
English	1 [Reference]	1 [Reference]	1 [Reference]	1 [Reference]
Spanish	0.83 (0.70-0.98)	0.83 (0.70-0.98)	0.52 (0.44-0.61)	0.55 (0.48-0.63)
No. of cancer family history records				
1	1 [Reference]	1 [Reference]	1 [Reference]	1 [Reference]
2	1.46 (1.36-1.58)	1.46 (1.35-1.57)	1.44 (1.35-1.54)	1.44 (1.36-1.53)
≥3	1.78 (1.62-1.96)	1.77 (1.61-1.95)	1.76 (1.60-1.94)	1.74 (1.60-1.90)
No. of first-degree relatives with cancer history				
0	1 [Reference]	1 [Reference]	1 [Reference]	1 [Reference]
1	1.23 (1.16-1.31)	1.23 (1.15-1.31)	1.16 (1.10-1.23)	1.15 (1.09-1.21)
≥2	1.64 (1.51-1.77)	1.64 (1.52-1.78)	1.33 (1.22-1.45)	1.35 (1.25-1.46)
No. of second-degree relatives with cancer history				
0	1 [Reference]	1 [Reference]	1 [Reference]	1 [Reference]
1	1.37 (1.28-1.47)	1.37 (1.27-1.47)	1.71 (1.60-1.82)	1.70 (1.61-1.81)
≥2	1.88 (1.72-2.05)	1.89 (1.73-2.06)	2.17 (1.96-2.39)	2.19 (2.00-2.39)
No. of first- or second-degree relatives with a rising mortality cancer				
0	1 [Reference]	1 [Reference]	1 [Reference]	1 [Reference]
1	3.85 (3.63-4.09)	3.84 (3.63-4.08)	2.80 (2.68-2.93)	2.76 (2.65-2.87)
≥2	3.73 (3.27-4.25)	3.66 (3.21-4.17)	2.51 (2.28-2.77)	2.40 (2.20-2.62)
Hereditary cancer-related term found in family history section comment				
No	1 [Reference]	1 [Reference]	1 [Reference]	1 [Reference]
Yes	2.00 (1.71-2.34)	2.00 (1.71-2.33)	1.91 (1.73-2.12)	1.91 (1.74-2.09)
Comprehensiveness score				
2	1 [Reference]	1 [Reference]	1 [Reference]	1 [Reference]
3	3.55 (3.32-3.79)	3.52 (3.29-3.77)	2.31 (2.20-2.42)	2.32 (2.23-2.42)

Abbreviations: APR, adjusted prevalence ratio; GARDE, Genetic Cancer Risk Detector.

^a^ Multiple imputation results were based on 20 datasets.

^b^ Includes American Indian or Alaska Native, Asian, Middle Eastern or North African, and Native Hawaiian or Other Pacific Islander.

For NYULH, where data were not MCAR, we observed notable differences between analytical approaches. Meeting criteria at NYULH was associated with older age (45-60 compared with 25-29 years: APR, 1.36 [95% CI, 1.22-1.51]), female sex (compared with male sex: APR, 1.27 [95% CI, 1.22-1.32]), higher cancer family history metrics (eg, ≥2 compared with 0 first-degree relatives with cancer history: APR, 1.35 [95% CI, 1.25-1.46]), presence of a hereditary cancer-related term in family history comments (compared with no presence: APR, 1.91 [95% CI, 1.74-2.09]), and higher comprehensiveness score (3 compared with 2: APR, 2.32 [95% CI, 2.23-2.42]). By contrast, not meeting criteria at NYULH was associated with Hispanic ethnicity (compared with non-Hispanic White race: APR, 0.81 [95% CI, 0.69-0.94]) and Spanish language preference (compared with English: APR, 0.55 [95% CI, 0.48-0.63]). Although most APRs and 95% CIs remained relatively similar, age (eg, 30-44, complete case analysis compared with multiple imputation analysis: APR, 1.58 [95% CI: 1.40-1.78] compared with 1.46 [95% CI, 1.31-1.62]) and number of first- or second-degree relatives with rising mortality cancers (≥2 compared with 0, complete case analysis compared with multiple imputation analysis: APR, 2.51 [95% CI, 2.28-2.77] compared with 2.40 [95% CI, 2.20-2.62]) showed the largest discrepancies. Specifically, complete case analysis yielded different association magnitudes for these variables compared with multiple imputation, with larger estimates observed in the complete case approach, suggesting that older individuals (compared with those aged 25-29 years) and those with first- or second-degree relatives with a rising mortality cancer (compared with individuals without first- or second-degree relatives with a rising mortality cancer) were more likely to meet criteria when missing data were discarded rather than imputed. This pattern of bias aligns with the MCAR test findings, confirming that the missing data mechanism at NYULH was not random. In addition, this finding suggests that the estimates generated from multiple imputation were more accurate than those from complete case analysis.

## Discussion

This investigation addressed 2 key research questions: (1) Can a CDS algorithm identify patients who meet criteria for hereditary cancer genetic evaluation when family history data are incompletely documented in the EHR? (2) Is data missingness associated with identification patterns in patient subgroups? We analyzed data from 2 large health care systems with different patterns of missing data. At UHealth, where data appeared to be MCAR, incomplete documentation was not associated with identification of eligible patients, regardless of patient subgroup. In contrast, we observed differences at NYULH, where missing data showed systematic patterns. Specifically, older patients and those with relatives who had rising mortality cancers were more likely to be identified as meeting criteria when incomplete records were excluded from analysis rather than when missing information was estimated.

These contrasting findings across health care systems suggest that the magnitude of the association between incomplete documentation and algorithmic identification of patients eligible for hereditary cancer genetic evaluation depends on whether data are missing randomly or systematically. When data are MCAR, as observed at UHealth, the probability of missingness is unrelated to both observed and unobserved patient characteristics (ie, any patient has an equal chance of having incomplete family history documentation regardless of their demographic or clinical factors). In contrast, systematic missingness, as observed at NYULH, occurs when the probability of having incomplete documentation is related to observed characteristics. In the current study, this manifested as older patients and those with relatives who had rising mortality cancers showing different patterns of identification depending on whether incomplete records were excluded or imputed. These results highlight the importance of understanding local documentation patterns when implementing CDS algorithms for cancer genetic evaluation, and they suggest that different approaches to handling missing family history data may be necessary across different health care systems.

The BRIDGE study team has investigated factors affecting cancer family history availability in the UHealth and NYULH health care systems^22^ and evaluated enhancements to the GARDE algorithms.^29,30^ The present study builds on this foundation by making a contribution to the CDS algorithm literature, demonstrating the association between missing data patterns and identification of patients who meet the criteria for hereditary cancer genetic evaluation. Future replication studies in other health care systems should harmonize unstructured and structured EHR data with information obtained directly from patients’ family members. This multisource approach has proven valuable.^31^ A validation study conducted in the Greater Cincinnati area in Ohio found that using a multiple-informant strategy identified an additional 7% to 13% of individuals who had been diagnosed with heart disease, type 2 diabetes, high cholesterol, or hypertension compared with a single-informant assessment.^31^

Prior research has shown that reporting and documentation of family history in the EHR vary by age, with older individuals more likely to have gaps in documentation.^32,33,34^ These previous studies have also shown that the completeness of family history of cancer may differ by cancer type.^32,34^ In one study, patients with certain cancer types (ie, breast or colorectal) were more likely to have a complete family history, but patients with prostate, pancreatic, or endometrial cancer were not.^34^ Although many barriers to the collection of family history information in health care settings have been found,^10,35,36,37^ the reasons for different patterns of missingness across systems have not been well studied. Our finding of different patterns in 2 health care systems suggests that in developing interventions to improve the completeness of family history documentation in the EHR, organization-level factors, such as clinical workflows and practitioner and clinical staff training, will be critical to examine further. Although alternative system-level approaches to genetic evaluation, such as population screening rather than risk-based identification of those meeting specific criteria for genetic evaluation, would not depend on the completeness of EHR data, this approach is not yet broadly implemented outside of research studies, and criteria for evaluation used in clinical settings are still based on personal and family cancer history.^18,30,38^

### Strengths and Limitations

The current investigation has several methodological strengths. We analyzed large samples from 2 distinct health care systems (n = 55 918 at UHealth and 101 289 at NYULH), overcoming the sample size limitations that have constrained previous studies relying on self-reported family history data.^39^ This retrospective analysis also benefited from combining structured EHR data elements with unstructured information extracted from free-text family history comments, capturing nuanced details that structured fields alone could miss.^40,41^ Additionally, we employed multivariate imputation by chained equations, which used all available variables to simultaneously estimate missing values, thereby accounting for nonignorable missing data.^42,43^

This study also has limitations. First, we did not extract unstructured data from other sources such as pathology results, clinical notes, and templated reports, which may have contained family history information not captured in the structured family history and associated comment sections we analyzed.^44^ Second, we excluded patients without any family history records, who represented more than 60% of the initial patient population at each site (61.3% at UHealth and 73.2% at NYULH) (Figure). This exclusion was necessary because regression estimates become unreliable beyond 40% missingness, making multiple imputation inappropriate at such high levels.^45^ This methodological constraint has important implications. Patients’ knowledge of family health history varies across several characteristics (eg, health literacy and educational attainment), and not all known information is communicated to clinicians or recorded in EHRs.^46,47,48,49,50^ In addition, missing family history records could have resulted from patient differences in health care engagement, cultural communication patterns, or mistrust of health care institutions.^5,6,7,8,22^ Consequently, our analysis could not assess meeting GARDE algorithm criteria for hereditary cancer risk for a considerable number of patients who may have been eligible, limiting the generalizability of our findings.

## Conclusions

Based on the comprehensive analysis of incomplete family history documentation in EHRs in this cross-sectional study, we found that the magnitude of the association between data missingness and identification of patients eligible for hereditary cancer genetic evaluation depended on whether data were missing randomly or systematically. At UHealth, where missing data patterns appeared random, incomplete documentation was not associated with identification of eligible patients across any demographic subgroup. In contrast, at NYULH, where data showed systematic missingness, older patients and those with relatives who had rising mortality cancers were more likely to be identified when incomplete records were excluded rather than imputed. It is also important to highlight that we did not find an association between incomplete documentation and identification of eligible patients across sex and race and ethnicity. These findings underscore that CDS algorithms for cancer genetic evaluation may perform differently across health care systems based on local documentation practices. Understanding the mechanisms of missing data is therefore critical when implementing such algorithms. Future research should aim to identify the underlying causes of these patterns, including patient-practitioner communication barriers, cultural factors that affect family health information sharing, and systematic differences in documentation practices. Health care organizations implementing CDS algorithms should assess their specific patterns of missing data and consider tailored approaches to handling incomplete family history information. This will help ensure equitable identification of all patients who could benefit from genetic evaluation services.

## Notes

Supplement.
Data Sharing Statement
