# Supplementary material for: Incomplete Family History and Meeting Algorithmic Criteria for Genetic Evaluation of Hereditary Cancer
Source: JAMA Netw Open. 2025 Oct 28;8(10):e2539870. doi: 10.1001/jamanetworkopen.2025.39870 (PMC12569706; doi:10.1001/jamanetworkopen.2025.39870)
Supplement: Supplement. — Data Sharing Statement [file jamanetwopen-e2539870-s001.pdf]

Data Sharing Statement

Harris. Incomplete Family History and Meeting Algorithmic Criteria for Genetic Evaluation of Hereditary Cancer. *JAMA Netw Open*. Published October 28, 2025. doi:10.1001/jamanetworkopen.2025.39870

Data
Data available: Yes
Data types: Deidentified participant data
How to access data: The data that support the findings of this study are available from the corresponding author upon reasonable request.
When available: With publication

Supporting Documents
Document types: None

Additional Information
Who can access the data: Researchers whose proposed use of the data has been approved.
Types of analyses: Specified purpose
Mechanisms of data availability: The data that support the findings of this study are available from the corresponding author upon reasonable request.
